# Glomerular Disease in Patients with Infectious Processes Developing Antineutrophil Cytoplasmic Antibodies

**DOI:** 10.5402/2013/324315

**Published:** 2013-02-19

**Authors:** Konstantin N. Konstantinov, Suzanne N. Emil, Marc Barry, Susan Kellie, Antonios H. Tzamaloukas

**Affiliations:** ^1^Division of Rheumatology, Department of Medicine, Raymond G. Murphy VA Medical Center, University of New Mexico School of Medicine, Albuquerque, NM 87131, USA; ^2^Department of Pathology, University of New Mexico School of Medicine, MSC08 4640, BMSB, Room 335, University of New Mexico, Albuquerque, NM 87131, USA; ^3^Division of Infectious Diseases, Department of Medicine, Raymond G. Murphy VA Medical Center, University of New Mexico School of Medicine, Albuquerque, NM 87131, USA; ^4^Division of Nephrology, Department of Medicine, Raymond G. Murphy VA Medical Center, University of New Mexico School of Medicine, VA Medical Center (111C), 1501 San Pedro, SE, Albuquerque, NM 87131, USA

## Abstract

To identify differences in treatment and outcome of various types of glomerulonephritis developing in the course of infections triggering antineutrophil cytoplasmic antibody (ANCA) formation, we analyzed published reports of 50 patients. Immunosuppressives were added to antibiotics in 22 of 23 patients with pauci-immune glomerulonephritis. Improvement was noted in 85% of 20 patients with information on outcomes. Death rate was 13%. Corticosteroids were added to antibiotics in about 50% of 19 patients with postinfectious glomerulonephritis. Improvement rate was 74%, and death rate was 26%. Two patients with mixed histological features were analyzed under both pauci-immune and post-infectious glomerulonephritis categories. In 9 patients with other renal histology, treatment consisted of antibiotics alone (7 patients), antibiotics plus immunosuppressives (1 patient), or immunosuppressives alone (1 patient). Improvement rate was 67%, permanent renal failure rate was 22%, and death rate was 11%. One patient with antiglomerular basement disease glomerulonephritis required maintenance hemodialysis. Glomerulonephritis developing in patients who became ANCA-positive during the course of an infection is associated with significant mortality. The histological type of the glomerulonephritis guides the choice of treatment. Pauci-immune glomerulonephritis is usually treated with addition of immunosuppressives to antibiotics.

## 1. Introduction

The reaction of the body to infections may cause secondary illnesses, such as rheumatic fever and poststreptococcal glomerulonephritis, with potentially life-threatening manifestations. An early step in the research to unravel the link between infection and secondary illness was the discovery that chronic infections trigger multiple immunological responses potentially associated with specific diseases [1]. Infections can lead to formation of multiple autoantibodies, for example, rheumatoid factor, antinuclear antibodies (ANAs), antiphospholipid antibodies, and antineutrophil cytoplasmic antibodies (ANCAs). The development of autoantibodies in the course of an infection may or may not be associated with manifestations of autoimmune disease. 

The recognition of autoimmune manifestations during the course of an infection and the decision to add specific treatment for the secondary immunological abnormality or to treat only the infection and monitor the patient for attenuation and disappearance of the immunological manifestations are critical tasks. The search for organ manifestations that can help the distinction between infectious and immunological manifestations can assist the diagnostic process. The kidneys, and particularly the glomeruli, are frequently affected by either the primary infectious process or the secondary immunological disease.

It has been known for a long time that glomerulonephritis may complicate a severe infection (endocarditis) [2]. Immune complex deposition in glomeruli, through either in situ formation [3] or trapping of circulating immune complexes [4], was the mechanism for glomerulonephritis following bacterial infections that was established first [5–7]. In one case of postinfectious glomerulonephritis, streptococcal antigens were found in the immune deposits in the kidney [8]. In a second case, antibodies against *Enterococcus* were eluded from the kidney [9]. 

This paper addresses glomerulonephritis associated with the formation of ANCA during the course of an infection. We identified published reports of ANCA formation during the course of infections and selected reports containing information about renal histology. We reviewed the histological types of renal lesions, the treatment of the renal disease, and the outcomes of the patients. Finally, we analyzed the pathogenesis of ANCA formation during the course of infections. 

## 2. ANCA Formation during the Course of Various Illnesses

ANCA can be formed following environmental exposure (silica), use of drugs, or during the course of various disease processes. Drugs most frequently leading to ANCA formation are hydralazine, propylthiouracil, penicillamine, allopurinol, and sulfasalazine [10]. The list of medical conditions which are associated with ANCA formation during their course is expanding, like the list of drugs. These conditions are classified into one of three categories [11]. (a) Chronic inflammatory processes: rheumatoid arthritis, inflammatory bowel disease, sweet syndrome, eosinophilia-myalgia syndrome, Goodpasture syndrome [11], systemic lupus erythematosus [12], mixed connective tissue disease [13], and chronic liver disease [14] including primary sclerosing cholangitis [15] and autoimmune hepatitis [16], (b) neoplasms: solid tumors (small cell lung carcinoma, renal carcinoma, colonic carcinoma, gastric carcinoma, pancreatic tumors, and thymoma), atrial myxoma, lymphoma, myeloma, myelodysplasias and hematopoietic stem cell transplantation [11, 17–22], and (c) infections. 

 Although the impact of ANCA formation and ANCA-associated disease on the course of each disease category is worth studying, the relation between infection and formation of ANCA deserves special emphasis. Two lines of evidence suggest a central role for infections in the formation of ANCA. (a) Various types of ANCA formation during infections, including ANCA directed at antigens other than myeloperoxidase (MPO) or proteinase-3 (PR-3), have been explored in the laboratory. This topic will be discussed in the section on pathogenesis. (b) Clinical studies suggest that formation of ANCA in several conditions considered noninfectious, such as sclerosing cholangitis, autoimmune hepatitis, inflammatory bowel disease [23], and rheumatoid arthritis [24] is triggered by secondary infections. 

The finding of ANCA positivity (as well as positive serologies for other autoantibodies such as antiphospholipid antibodies, antinuclear antibodies, etc.) during the course of an infectious process generates two critical questions. (a) Is the autoantibody only a serological test without any other clinical significance or autoantibody-mediated disease complicates the infection? The distinction is difficult because infections and immunological disease share several frequent manifestations. A careful investigation for manifestations caused by autoantibodies is warranted. (b) If clinical manifestations of autoimmune disease are found, will treatment of the infection also reverse the autoimmune manifestations, or additional measures directed towards these manifestations are needed? This constitutes a therapeutic dilemma. Autoimmune manifestations may be life threatening, but on the other hand, methods of treatment of autoimmune diseases are associated with increased risks of sepsis [25–27]. 

Study of the kidneys should be targeted in patients with infections and ANCA positivity because kidneys are one of the main organs affected by ANCA-associated disease. Of note is that kidneys may be affected by various pathogenetic mechanisms during the course of severe infections. In one study, the histological patterns of renal disease associated with infectious endocarditis included in descending frequency renal infarcts, approximately one-half of which were due to septic emboli, glomerulonephritis, acute interstitial nephritis, and cortical necrosis [28]. In addition, the histological picture of glomerulonephritis varies. Glomerulonephritis with immune complex deposition characterizing the classical postinfectious variety, or IgA nephropathy, and vasculitis/pauci-immune glomerulonephritis have all been described [29]. 

## 3. Infectious Processes Associated with Formation of ANCA

We reviewed reports of ANCA formation during the course of infections found in a PubMed search and reports found in the reference lists of the PubMed reports. We found four types of reports, laboratory studies analyzing large, usually, numbers of sera from patients with a specific infection, reviews, epidemiological studies, and clinical reports presenting details of one case or a small number of cases. 

Tables 1 and 2 show categories of infectious agents associated with ANCA positivity. For infectious agents with multiple reports of ANCA association, for example, human immunodeficiency and hepatitis viruses, we selected representative articles for the list of references. The list of infections associated with ANCA formation will, in all probability, expand. New associations are continuously reported. It is probable that a few associations included in Tables 1 and 2 represent superinfection in patients with primary ANCA-associated disease, rather than ANCA formation during the course of an infection. Finally, it is possible that we missed some reported infections associated with formation of ANCA despite a systematic search. 

## 4. Glomerulonephritis in Patients with ANCA Formation during Infectious Episodes

We present a synthesis of published reports of infectious episodes with ANCA formation and known renal histology. These reports belong almost exclusively in the case report category. There is a paucity of systematic studies analyzing substantial numbers of patients. Therefore, the impact of ANCA formation during the course of an infectious process with glomerular involvement on the course of the renal disease, the choice of treatment, and the outcome of the patients will require further studying. It is possible that a minority (two or three) of the reports analyzed represent superinfection in patients with primary ANCA-associated disease.

We found 35 references reporting 50 patients with histologically documented glomerular disease and ANCA positivity or renal vasculitis/pauci-immune glomerulonephritis with negative ANCA serology during the course of infectious episodes. Twenty references [35, 36, 40, 51, 52, 103–107, 109, 111, 113–115, 123–127] reported 27 patients with infective endocarditis, while 15 references [39, 50, 62, 63, 108, 110, 112, 116–122, 128] reported 23 patients with other infectious episodes. Age of the patients varied between 6 and 87 years (endocarditis 6–87, mean ± standard deviation 44.7 ± 21.1 years, other infections 17–74, 48.7 ± 16.0 years). Of the 50 patients, 18 (36%) were female and 32 (64%) were male. There was a preponderance of male patients among subjects with endocarditis (males 20 of 27, or 74%, females 7 of 27, or 26%), while the percentages of females (11 of 23, or 48%) and males (12 of 23, or 52%) were similar in patients with other infections. 

Table 3 shows the microbial etiology of the infections. Blood cultures revealed more than one species of micro-organisms in several patients [51, 63, 108]. Three species of bacteria, *Streptococcus, Staphylococcus*, and *Enterococcus*, accounted for 63% of the reported episodes of infectious endocarditis and 42% of all infections reported. In patients with endocarditic due to *Bartonella* [113, 114] and *Brucella* [115], the diagnosis was not made by blood cultures, which were negative, but by clinical examination, echocardiography, serologic tests, and response to antibiotics. Infectious endocarditis, in the absence of positive blood cultures, was diagnosed by histological examination of cardiac valves in four patients [123, 126, 127] and [124, (b)]. Endocarditis in the absence of positive blood cultures was diagnosed by clinical evaluation, echocardiographic findings, and the response to antibiotics in two patients [124, (a)] and [125]. The evolution of clinical and laboratory status under antibiotic treatment was used to diagnose an active infection other than endocarditis in one patient [114]. 

## 5. Laboratory Tests in Patients with Infections, ANCA Positivity, and Glomerulonephritis

Table 4 shows abnormalities in the laboratory tests in the 50 patients analyzed in this report. Anemia was almost universal. The only hematological difference between patients with endocarditis and those with other infections was a slightly higher mean white blood count in those with other infections. Among tests indicating inflammation, blood erythrocyte sedimentation rate and serum C-reactive protein were almost universally elevated, while depression of serum complement components was found in approximately half of the patients.

A variety of autoantibodies was reported with no appreciable differences between patients with endocarditis and those with other infections. ANCA positivity without any identification of ANCA specificity was reported in 9 patients [50, 121]. The specificity of ANCA changed from p-ANCA to c-ANCA during the course of the disease in one patient [35]. Another patient had both anti-PR-3 and anti-MPO ANCA [107]. Finally, ANCA with specificities other than MPO or PR-3 was found in two instances [62, 112]. These findings are relevant to the discussion of formation of ANCA with various specificities during the course of infectious episodes which will follow later in this paper. 

Tests less frequently reported included serum antiphospholipid antibody, which was positive in 6 of 8 patients (75%) with endocarditis and negative in one patient with infection other than endocarditis, polyclonal elevation of immunoglobulins reported in 12 patients with endocarditis and 4 patients with other infections, and circulating immune complexes detected in 4 patients with endocarditis and 3 patients with other infections. Antiglomerular basement membrane antibody was positive in one of the five patients tested (20%).

## 6. Renal Manifestations in Patients with Infections, ANCA Positivity, and Glomerulonephritis

Table 5 summarizes urinary and renal function findings in these patients. Reports presenting more than 3 patients tended to omit details in renal function [36, 50]. Hematuria was almost universal. The low number of patients with dysmorphic red cells or red cell casts may reflect performance of urinalysis by automated means in a number of the instances. Pyuria was probably underreported. Some degree of proteinuria was found in all studied patients. Approximately, one third of the patients had nephrotic proteinuria. The degree of proteinuria was the only identified difference between patients with endocarditis and those with other infections. Patients with infections other than endocarditis appeared to have a higher frequency of nephrotic proteinuria. Cryoglobulinemic glomerulonephritis secondary to hepatitis C may have contributed to this finding [120]. Serum creatinine was elevated in 95% of the patients. Advanced renal failure requiring temporary or permanent dialysis was reported in 18% of the patients. It is probable that as high as 24% required dialysis.

Table 6 shows the types of renal pathology and their association with hypocomplementemia and various ANCA specificities. The classification of glomerular lesions was done by two of the authors of this report (Barry and Tzamaloukas). In a small number of cases, our characterization of the glomerular lesion differed from that of the authors of the original reports. The classification encountered the following difficulties which should be noted because they may guide future efforts. (a) Immunofluorescence and, even more frequently, electron microscopy findings, essential for the diagnosis of the type of glomerulonephritis, were missing from many reports. (b) Renal pathology in a few instances was done on autopsy material, which may have produced false negative immunofluorescence findings. (c) Information which would provide convincing evidence for a histological diagnosis was missing from several reports. (d) Several patients had a combination of features of pauci-immune glomerulonephritis and features of postinfectious glomerulonephritis with immune complex deposition. This last feature merits a few comments.

The concept of “dual glomerulopathy” denotes deposition of immune complexes in the kidneys of patients with ANCA-associated pauci-immune glomerulonephritis [129, 130] or development of ANCA-associated pauci-immune glomerulonephritis or vasculitis in patients with immune complex-mediated glomerulonephritis [131–133]. Two of the cases analyzed in this report [120, (a) and (b)] were classified by both the authors of the report and the authors of this paper as having “dual glomerulopathy” and were classified under both “pauci-immune GN” and “postinfectious GN” categories in Tables 6 and 7. Another two cases, classified as pauci-immune glomerulonephritis or vasculitis [112, 123], probably had dual glomerulopathy. 

ANCA formation in patients with postinfectious glomerulonephritis has been reported [134]. Whether the development of ANCA positivity without renal histological development of ANCA-mediated disease in these patients affects the severity of the renal disease remains an open question. One report found no effect [135], while another report found higher rate of crescent formation in the glomeruli of patients with postinfectious glomerulonephritis who became ANCA-positive [136]. However, the superimposition of ANCA-associated renal lesions on immune complex-mediated glomerulonephritis in patients without active infections worsens the severity of renal manifestations [130, 137, 138].

**Figure 1:**
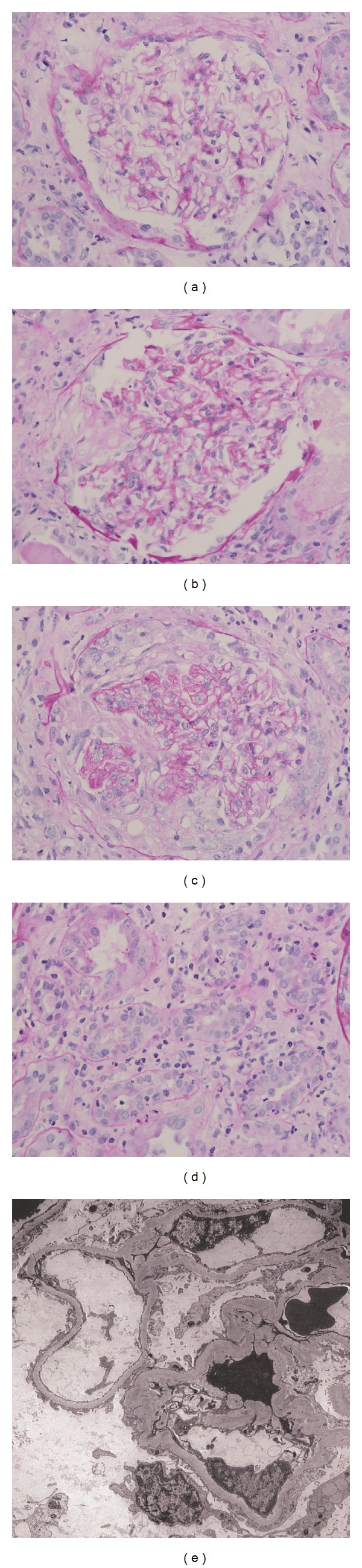
Pauci-immune glomerulonephritis and acute interstitial nephritis in a patient with ANCA triggered by infectious endocarditis. (a) Normal appearing glomerulus without endocapillary or mesangial proliferation (PAS; 400x). (b) Glomerulus with area of segmental scarring: nonscarred portion of tuft appears to be within normal limits (PAS; 400x). (c) Glomerulus with crescent formation (PAS; 400x). (d) Tubulointerstitial inflammation (PAS; 400x). (e)–(EM): Portion of tuft without significant electron densities, and without endocapillary or mesangial hypercellularity (5000x).

**Figure 2:**
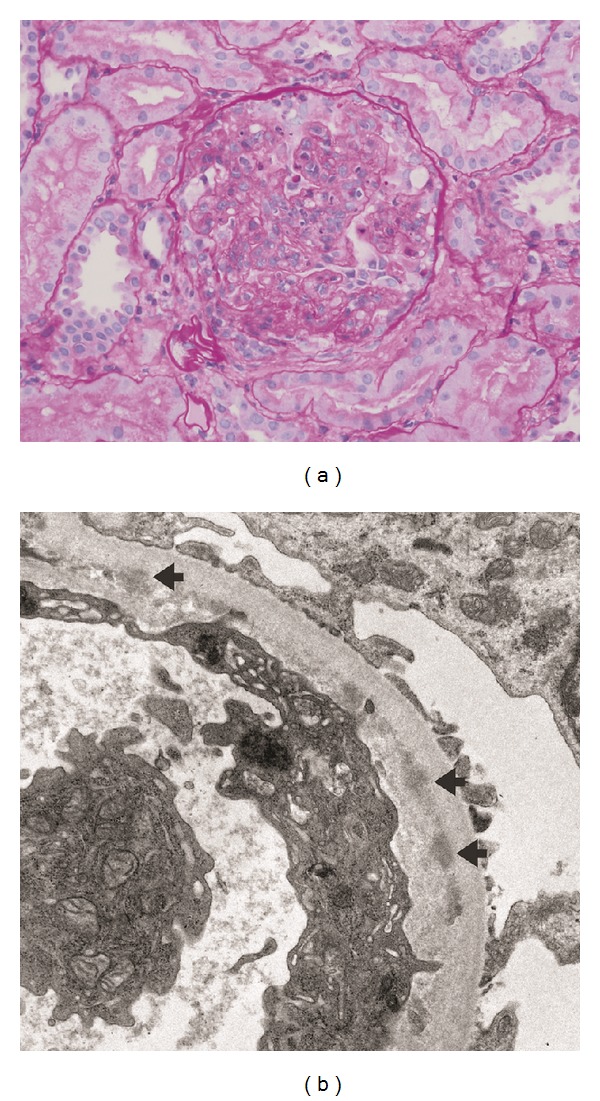
Postinfectious glomerulonephritis. (a) Glomerulus showing diffuse endocapillary proliferation, including intracapillary neutrophils (400x). (b)–(EM): Electron micrograph of a glomerular peripheral capillary loop showing subendothelial electron dense deposits (arrows) (6000x).

Pauci-immune and postinfectious glomerulonephritis were the most frequent histological diagnoses in Table 6. Figure 1 shows a pauci-immune glomerulonephritis developing a patient with bacterial endocarditis. Figure 2 shows a postinfectious glomerulonephritis. A few other findings in Table 6 are worth noticing. Some patients had more than one renal lesion (e.g., dual glomerulopathy or combination of glomerulonephritis and acute interstitial nephritis with features of tubulitis) and were classified in more than one row in this table. One patient with human immunodeficiency (HIV) infection and multiple autoantibodies including ANCA and antibasement membrane (anti-GBM) antibodies developed anti-GBM glomerulonephritis with linear deposition of IgG and C_3_ on the glomerular basement membrane [121]. Anti-GBM glomerulonephritis has been reported in two other patients with HIV infection [139, 140]. Finally, hypocomplementemia and positivity with c-ANCA and/or anti-PR-3 antibody were noted more frequently in patients classified in the postinfectious glomerulonephritis than those classified in the pauci-immune glomerulonephritis category. Whether this difference can be used as a differential diagnosis clue should be a subject of future studies.

## 7. Diagnosis and Differential Diagnosis

The diagnosis of ANCA-associated disease superimposed on an infection encounters obstacles. The first obstacle is the similarity of the clinical manifestations between the two categories of diseases. Malaise, weakness, fever, anorexia, weight loss, arthralgias, and myalgias are cardinal manifestations of both disease categories as are several laboratory findings including anemia, leucocytosis, and thrombocytopenia. Certain localized infections cause manifestations that may be indistinguishable from those of ANCA-associated disease. For example, auscultatory and echocardiographic features of infectious endocarditis are essentially the same as those of endocarditis caused by involvement of the cardiac valves by granulomatosis with polyangiitis [141–150]. Given the diagnostic difficulties, awareness about the possibility of formation of ANCA and other autoantibodies and performance of appropriate serological tests during the course of infections with unusual clinical features is the first step in the differential diagnosis. 

The second major diagnostic difficulty is that patients with severe or complicated infections often develop multiple autoantibodies (Table 4). Autoantibodies that may become positive during infectious episodes and can be associated with specific renal diseases include, in addition to ANCA, antinuclear antibodies, antiphospholipid antibodies, antiglomerular basement membrane antibodies, and cryoglobulins. For example, HIV infection triggers the formation of multiple autoantibodies, which is associated with a substantial variety of renal diseases, and causes major diagnostic difficulties [151–153]. The diagnostic difficulties in HIV positive patients are not limited to the kidneys. Manifestations in several other organs, for example, the lungs [154], create similar diagnostic difficulties.

Given the high frequency of renal involvement during serious infectious episodes and the variety of renal pathologies that can be seen in patients with infections, renal function should be monitored with frequent urine examinations and measurements of serum creatinine concentration. If renal involvement is diagnosed, kidney biopsy with complete histological evaluation by light microscopy, immunofluorescence, and electron microscopy should be performed in a timely manner [153]. The search for exact histological diagnosis of the renal disease in patients with infections and a multiplicity of autoantibodies is important for understanding the biology of infectious processes but begs the question whether knowledge of the nature of the renal lesion will affect the management and outcome of the patient. The next section of this report addresses this issue.

## 8. Treatment and Outcome

The view that adequate treatment of the infectious process will result in cure of both the infection and the secondary immunological abnormalities without any other intervention has been expressed repeatedly. It can be argued, however, that serious manifestations mediated by autoantibodies triggered by infections require specific treatment. This second view should not be addressed in a trivial manner when ANCA-associated disease is found. The immunosuppressive measures employed in the treatment of ANCA-associated disease carry several risks, among which infection and sepsis are arguably the most important [155–157]. If these measures are applied during the course of severe infections, the risks of sepsis and septic death could increase substantially. To determine whether there is a place for measures directed against ANCA-associated disease in patients with infections triggering ANCA-associated glomerulonephritis, this section analyzes the treatment and outcome of the 50 patients with infections, positive ANCA and glomerular pathology. 

Table 7 shows treatment, duration of followup, and outcomes of the 50 patients analyzed in this report. All but one [108] patient with pauci-immune glomerulonephritis received immunosuppressive therapy or plasma exchange. One patient [123] had cardiac valve replacement surgery. The response to treatment was not reported in three patients [52, 110] and [128, (b)]. Followup of the remaining 20 patients ranged between one week and 11 years. Among these patients, 17 (85%) showed improvement of their clinical status or complete recovery, while ANCA, not reported in 16 patients, became negative during followup in 4 subjects (17%). Prompt clinical response to plasma exchange [104] or cyclophosphamide plus corticosteroids [107] was noticed in two patients whose clinical picture was deteriorating, while they were receiving only antibiotics. Two other patients with hepatitis C and combination of immune complex glomerulonephritis and vasculitis [120, (a) and (b)] were initially treated with corticosteroids. Clinical picture and renal function improved only after addition of alpha interferon in the first patient and plasma exchange in the second. Two deaths occurred one week and 12 days after treatment with corticosteroids only or antibiotics only. It appears that death was secondary to disseminated vasculitis in the first patient [127] and to atherosclerotic coronary artery disease in the second patient [108]. Another patient, in whom the clinical and laboratory response to the treatment with corticosteroids and cyclophosphamide was not reported, died six months after diagnosis from a small bowel infarction [110]. It is not clear whether the bowel infarction was the result of vasculitis or other disease of the vessels.

Antibiotics were administered to 17 of the 19 patients with postinfectious glomerulonephritis, while 11 patients received also corticosteroids. Followup, not reported in one patient, ranged between 1 month and 4 years in the remaining 18 subjects. ANCA titer decreased or became negative in 11 patients (59%), remained positive after 5 months in one patient (5%) with improved clinical status, and was not reported in the remaining 7 patients. Aortic valve replacement was performed in 2 patients [111, 126], while a third patient [106] had combined aortic and mitral valve replacement. Three patients had removal of infected atrioventricular shunts and recovered promptly after surgery [116, 118, 122]. One patient [105] had repair of a ventricular septal defect four years after successful treatment. Clinical improvement or complete recovery was noted in 14 of the 19 patients (74%). Four of the five deaths [36, (d)], [40, (b)], and [106, 125] occurred within two months of the diagnosis, while the time of death for the other patient [111] was not reported. Death was caused by cardiac disease in two patients [40, (b)] and [125], one of whom was on maintenance hemodialysis [125] and intracerebral hemorrhage in another patient on hemodialysis [106]. The cause of death was not reported in two patients [36, (d)] and [111]. Renal function improved in the third patient on hemodialysis [126], who was able to discontinue this treatment. 

All but one patient with renal histology not diagnostic of either pauci-immune or postinfectious glomerulonephritis received antibiotics. Followup ranged between 6 weeks and 2.5 years. The patient who did not receive antibiotics [62] was treated successfully with corticosteroids, cyclophosphamide, and methotrexate. One patient [36, (g)] died 2.5 months after diagnosis, a second patient [36, (e)] developed chronic renal failure, and a third patient [36, (f)] developed end-stage renal failure. Improvement of the clinical status was noticed in seven of the nine patients. ANCA titers, not reported in 3 patients, became negative in 4 of the 6 remaining patients (67%) and remained positive in 2 (33%). The patient with HIV infection and anti-GBM glomerulonephritis required maintenance dialysis [121]. 

Several conclusions can be drawn from Table 7. (a) The development of ANCA positivity and glomerulonephritis in patients with infectious episodes is associated with significant morbidity (renal failure in this case) and mortality, regardless of the renal histology. (b) Pauci-immune glomerulonephritis can be treated by immunosuppressive regimens successfully and without increased incidence of sepsis. This conclusion should be interpreted with caution. The association between ANCA-mediated disease and infection with arbovirus [50] remains tentative. In addition, it is possible that the number of the patients analyzed had flares of primary ANCA-associated disease triggered by infection. Despite these reservations, treatment directed specifically against ANCA-associated disease should be given serious consideration for patients with infections, ANCA positivity, and pauci-immune glomerulonephritis or histological evidence of ANCA-associated disease in other organs. Surveillance for sepsis should be intense in patients treated with immunosuppressive regimes. 

(c) Antibiotic regimen targeted against the micro-organism responsible for the infection is the mainstay of management of postinfectious glomerulonephritis and is effective in most instances [158]. Corticosteroids may be added to the antibiotic regime. The efficacy and safety of adding corticosteroids in improving the renal outcome of postinfectious glomerulonephritis have not been studied in a prospective fashion. However, there are small retrospective studies [159] or case reports [160, 161] supporting addition of corticosteroids in such cases. The outcomes of the patients reported in Table 7 support this conclusion. 

(d) Removal of infected foreign bodies (atrioventricular shunts) or infected cardiac valves hastens recovery of both clinical and renal status. (e) Death rate appears to be higher in patients with postinfectious than those with pauci-immune glomerulonephritis. This last finding will require further studies. Factors affecting the outcomes of severe infections, such as differences in the underlying comorbidities and in the virulence of the causative infectious agents, or other factors were not studied in the reports analyzed. Studies of large numbers of patients who develop ANCA positivity and glomerulonephritis during the course of infectious episodes are needed. In addition to the histological type of the glomerulonephritis and the method of treatment, such studies should analyze other factors potentially affecting the outcomes of severe infectious episodes. 

## 9. Role of Infections in the Pathogenesis of ANCA-Mediated Disease

A body of evidence supports the view that ANCA is directly involved in the pathogenesis of pauci-immune glomerulonephritis and various vasculitides [162–165]. Infusion of anti-PR-3 ANCA into mice with humanized immune system produced vasculitic and renal lesions similar to those of granulomatosis with polyangiitis [166]. Current thinking about the role of ANCA in the pathogenesis of vasculitis is centered on activation of neutrophils by inflammatory cytokines and expression of ANCA antigens (PR-3, MPO, etc.), which are normally intracellular, on the surface of the “primed” neutrophils, where circulating ANCA can bind them. This binding activates the neutrophils and causes their adherence to vessel walls, release of proteolytic enzymes and oxygen radicals and subsequently endothelial activation, thrombin generation, and inflammatory injury to the wall of the blood vessels [167]. 

Epidemiological studies found an association between chronic nasal carriage of *Staphylococcus aureus* and granulomatosis with polyangiitis [168–170]. Chronic low-grade inflammation in the nasal cavity with release of proinflammatory cytokines, which cause priming of monocytes, has been proposed as a mechanism of triggering granulomatosis with polyangiitis by staphylococcal nasal carriage [171]. Immunization with bacterial proteins derived from *Staphylococcus aureus* caused ANCA formation and vasculitis in rats [172]. Infections cause both de novo ANCA formation with vasculitic lesions and relapses of established ANCA-associated disease [68, 173]. Finally, the role of prophylactic antimicrobial regimes, mainly trimethoprim-sulfamethoxazole, in preventing relapses of granulomatosis with polyangiitis has been established [174–178].

Staphylococcal superantigen, which had been proposed as the trigger for ANCA formation, does not appear to play that role [179]. The risk for relapse of granulomatosis with polyangiitis is higher in patients carrying staphylococcal strains producing toxic-shock-syndrome-toxin-1 [180]. Patients with ANCA-associated vasculitis harbor antibodies to both PR-3 and complementary PR-3 (cPR-3), which is a peptide translated from the antisense DNA strand of PR-3 [181, 182]. Several species of pathogenic micro-organisms, including *Staphylococcus aureus*, *Entamoeba histolytica*, and Ros river virus, have proteins with homology with cPR-3 [183]. 

Infections trigger formation of ANCA against a variety of cytoplasmic antigens, in addition to the two classical antigens PR-3 and MPO. A soluble protein in the neutrophil granules, bactericidal/permeability-increasing protein (BPI), has a role in the defense against Gram-negative bacteria. Anti-BPI autoantibodies were detected in a fraction of sera positive for ANCA by immunofluorescence and negative for MPO or PR-3 by ELISA assay [184]. Other antigens that are sources of autoantibodies recognized by immunofluorescence ANCA assay include azurocidin, which appears to be related to ANCA secondary to drugs [185], lactoferrin [185], and so forth. Anti-BPI ANCA is detected with a high frequency in patients with cystic fibrosis and infections with *Pseudomonas* species [53–56] and those with inflammatory bowel disease or primary sclerosing cholangiitis and presumed infections from enteric Gram-negative bacteria [186]. Anti-BPI ANCA increases the severity of both pulmonary and gastrointestinal infections through reductions of the clearance of bacteria and endotoxin by the leucocytes and, probably, through other nonspecific effects on immune complexes [187]. Whether anti-BPI ANCA is involved in the pathogenesis of vasculitis has not been clarified. The frequency of positivity for anti-BPI ANCA does not differ between patients with pauci-immune glomerulonephritis and those with other types of glomerular disease [188]. This finding indirectly suggests that anti-BPI ANCA does not have an important role in the pathogenesis of vasculitis. 

Another ANCA developing in infections is an autoantibody against human lysosome-mediated membrane protein-2 (LAMP-2). LAMP-2 is expressed on the membrane of the intracellular vesicles that also contain MPO and PR-3 and on the cell membrane of resting neutrophils and endothelial cells. ANCA against LAMP-2 cross-reacts with an adhesive fimbrial protein (FimH) of several species of Gram-negative bacteria. This findings suggest that molecular mimicry may be a mechanism of ANCA formation [189]. A study found that anti-LAMP-2 causes disease similar to human ANCA-mediated disease in experimental animals [189]. This finding was thought to provide a new insight into the pathogenesis of ANCA-mediated diseases [190, 191]. However, another study found the same frequency of anti-LAMP-2 ANCA in patients with pauci-immune glomerulonephritis and those with other types of glomerulonephritis and failed to show that anti-LAMP-2 ANCA causes vasculitic lesions or glomerulonephritis in experimental animals [192]. Thus, although the LAMP-2 pathway remains promising, more studies are needed to clarify its role as a mechanism in ANCA-associated disease and as a link between infection and pathogenic ANCA formation [193].

Another mechanism linking infection and ANCA is through production of neutrophil extracellular traps (NETs). NETs formation, which is an important mechanism for defense against bacterial infections [194], results in a unique type of neutrophil death (NETosis), with production of chromatin webs covered by proteins, including MPO and PR-3, that trap and kill microbes [195]. Several factors, including M1 protein, lipopolysaccharides, and bacteria such as *Streptococcus*, *Staphylococcus*, and *Enterococcus*, which have been found with high frequencies of ANCA formation during infectious episodes (Table 3), trigger NETs formation. Contact between ANCA IgG and primed neutrophils induces formation of NETs which stick to the endothelium and cause tissue damage [196]. Both PR-3 and NETs containing elastase were detected in glomeruli of patients with ANCA-associated vasculitis and acute worsening of the renal function, who had prominent neutrophil infiltrates in their kidneys. In addition, patients with ANCA-associated vasculitis, but not control subjects, had circulating MPO-DNA complexes [196]. These findings led to the hypothesis that ANCAs perpetuate a vicious NET production maintaining the delivery of antigen-chromatin complexes to the immune system. The role of infection in this scheme is to induce NETs formation [196, 197]. The study of NET formation in vasculitis [196] did not contain subjects with infections. Consequently, both the NETs hypothesis and the role of infection in it will need further study.

Another potential link between infections and ANCA formation is through toll-like receptors (TLRs) which sense pathogen-associated molecular patterns (PAMPs) such as bacterial cell wall proteins and bacterial DNA [198]. Various cell types, including neutrophils, monocytes, b-lymphocytes, endothelial cells, and epithelial cells express TLRs. TLR ligation causes release of proinflammatory cytokines, proliferation of lymphocytes, and production of antibody. In an *in vitro* study, stimulation of TLR9 by CPG, which are hypomethylated motifs prevalent in the DNA of bacteria, triggered production of ANCA from b-lymphocytes of patients with ANCA-associated vasculitis in remission [199]. In another study, the expression of TLR2, TLR4, and TLR9 in monocytes and natural killer cells was greater in patients with ANCA-associated disease than in healthy controls [200]. Thus, it is probable that TLRs play a role in the activation and/or relapse of ANCA-associated disease by infections. This mechanism also will need to be studied further.

Recent developments have focused on the genetics of ANCA-associated disease. Gene polymorphism and its relation to ANCA have been studied. The Z allele, but not the S deficiency allele of *α*_1_ antitrypsin, was associated with ANCA-associated vasculitis [201]. It was postulated that polymers of *α*_1_ antitrypsin, which were found in the serum and glomeruli of some patients with the Z allele, may promote inflammation by priming neutrophils [201]. Two other studies found associations of different types of ANCA-associated vasculitis with distinct gene variations. The first study found that both major histocompatibility-complex (MHC) and non-MHC are associated with ANCA-associated vasculitis and also that anti-PR-3 ANCA is associated with HLA-DP and the genes encoding *α*_1_ antitrypsin and PR-3, while anti-MPO ANCA is associated with HLA-DQ [202]. The second study found that the PTPN22 R620W allele is associated with granulomatosis with polyangiitis, especially its anti-PR-3 positive subset, but not with microscopic polyangiitis or Churg-Strauss syndrome [203]. The interaction of genetic and infectious influences on ANCA formation and ANCA-mediated disease will be a new research frontier [204]. Given the association between environmental factors (exposure to silica) and ANCA-associated disease, the interaction of epigenetic and infectious factors in the pathogenesis of ANCA formation and ANCA-mediated disease could also constitute a major research focus.

## 10. Conclusions

Glomerulonephritis developing in patients who became positive for ANCA during the course of infectious episodes is associated with morbidity and mortality, regardless of its specific histological picture. It poses profound diagnostic and therapeutic challenges. Monitoring of serum creatinine, urine sediment microscopy, and proteinuria in patients with protracted or severe infections and performance of a kidney biopsy if there is an indication of kidney injury are essential steps of the diagnostic process. Evaluation of the kidney biopsy must be thorough with light microscopy, immunofluorescence, and electron microscopy components. The diagnosis of ANCA-associated glomerulonephritis poses the critical question whether specific treatment directed towards ANCA-associated disease, with its inherent risks in patients with active infections, is required or not. Evaluation of the published reports suggests that there is a place for immunosuppressive regimen in the management of patients with infections who develop ANCA-associated glomerulonephritis or vasculitis. Determination of the exact indications for this immunosuppressive regimen will require prospective studies of large numbers of patients. The association between infections, development of ANCA positivity, and development of ANCA-mediated disease is an open field for research in fundamental mechanisms of immunology and in the derangements of these mechanisms.

## Figures and Tables

**Table 1:** Viral and bacterial infections associated with ANCA formation.

Virus species	References	Bacterial species	References
*Human immunodeficiency virus *(HIV)	[30–33]	*Streptococcus *	[34–36]
* Hepatitis B virus *	[37, 38]	*Staphylococcus *	[36, 39, 40]
*Hepatitis C virus *	[41–44]	* Enterococcus *	[36, 40]
* Parvovirus B-19 *	[45–48]	* Bartonella *	[49]
* Epstein-Barr virus *	[46]	* Gemella *	[49]
* Arbovirus *	[50]	* Propionibacterium *	[49]
		* Neisseria *	[51]
		* Actinobacillus *	[52]
		* Pseudomonas *	[53–56]
		* Escherichia *	[57]
		* Bacteroides *	[57]
		* Campylobacter *	[58]
		* Helicobacter *	[59]
		* Yersinia *	[58, 60]
		* Salmonella *	[60]
		* Corynebacterium *	[61]
		* Stenotrophomonas *	[62, 63]
		* Klebsiella *	[63]
		* Mycoplasma *	[64]
		* Chlamydia *	[65–67]
		* Rickettsia *	[68–70]
		* Treponema *	[71]
		* Leptospira *	[72]
		* Mycobacterium *	[73–82]

**Table 2:** Fungal, protozoal, and multicellular parasitic infections associated with ANCA formation infections associated with ANCA formation.

Category of infectious agent	References
Fungus species	
* Aspergillus *	[83–86]
* Histoplasma *	[87]
* Sporothrix *	[88]
* Pneumocystis *	[86, 89, 90]
* Paracoccidioides *	[91]
* Saccharomyces *	[92, 93]
Protozoa	
* Ameba *	[94, 95]
* Plasmodium *	[96–98]
* Leishmania *	[99]
Multicellular parasites	
* Echinococcus *	[100]
* Strongyloides *	[101]
* Toxocara *	[102]

**Table 3:** Microbial species associated with ANCA formation and glomerulonephritis in 50 patients.

Microbial species	Endocarditis references	Other infections references
*Streptococcus* species	[35], [36, (a)–(c)] [51, 103–107]	[108]
*Staphylococcus* species	[36, (e)-(f)], [109]	[39, 110]
*Enterococcus* species	[36, (d)], [40, 111]	[63]
*Actinobacillus actinomycetemcomitans*	[52]	[112]
*Bartonella* species	[113, 114]	
*Neisseria subflava *	[51]	
*Brucella* species	[115]	
*Stenotrophomonas* species		[62, 63]
*Propionibacterium* species		[116, 117]
*Klebsiella* species		[63]
*Gemella morbillorum *		[118]
*Escherichia coli *		[110]
*Mycoplasma *		[119]
*Arbovirus *		[50, (a)–(h)]
*Hepatitis C virus *		[120, (a) and (b)]
*Human immunodeficiency virus *		[121]
*Varicella-zoster virus *		[122]

Letters following the number of a reference denote patients in reports containing more than one patient.

**Table 4:** Abnormal laboratory tests in 50 patients with infections, ANCA positivity, and glomerular disease.

Laboratory test	Number of patients tested	Percent of tests with abnormality
Hematology		
Anemia^1^	23 (E: 16, O: 7)	96 (E: 100, O: 86)
Leucocytosis^2^	31 (E: 14, O: 17)	45 (E: 29, O: 59)
Leucopenia^3^	31 (E: 14, O: 17)	13 (E: 21, O: 6)
Shift to the left^4^	11 (E: 8, O: 3)	64 (E: 63, O: 67)
Thrombocytopenia^5^	17 (E: 13, O: 4)	24 (E: 23, O: 25)
Tests of inflammation		
Elevated Erythrocyte sedimentation rate^6^	22 (E: 8, O: 14)	100
Elevated C-reactive protein	28 (E: 19, O: 9)	93 (E: 100, O: 78)
Low C_3_ complement component	32 (E: 19, O: 13)	56 (E: 63, O: 46)
Low C_4_ complement component	35 (E: 22, O: 13)	46 (E: 45, O: 46)
Low C_H50_ complement component	20 (E: 9, O: 11)	60 (E: 67, O: 55)
Serum autoantibodies		
Positive antinuclear antibody	27 (E: 16, O: 11)	33 (E: 44, O: 18)
Positive cryoglobulins	19 (E: 11, O: 8)	32 (E: 36, O: 25)
Elevated rheumatoid factor	19 (E: 11, O: 8)	68 (E: 73, O: 63)
Positive c-ANCA^7^	33 (E: 24, O: 9)	64 (E: 63, O: 67)
Positive antiproteinase-3 (anti-PR-3)^8^	35 (E: 24, O: 11)	60 (E: 71, O: 36)
Positive p-ANCA^7^	35 (E: 25, O: 10)	31 (E: 32, O: 30)
Positive antimyeloperoxidase (anti-MPO)^8^	36 (E: 24, O: 12)	39 (E: 38, O: 42)

E: endocarditis, O: Other infections. ^1^Blood hemoglobin (Hgb) < 13 gm/dL. No difference between E and O. For all 27 subjects, mean Hgb was 8.4 ± 2.5 gm/dL. ^2^White blood cell count (WBC) > 10 k/mm^3^. ^3^WBC < 4 k/mm^3^. Mean WBC: E: 9.7 ± 6.8 k/mm^3^; O: 12.1 ± 8.5 k/mm^3^. ^4^Neutrophil predominance and/or immature white blood cells in WBC. ^5^Platelet count < 100 k/mm^3^. No difference between E and O. For all 17 subjects, mean platelet count was 160 ± 85 k/mm^3^. ^6^Mean erythrocyte sedimentation rate, not different between E and O, was in the 22 patients 77 ± 29 mm/hr. ^7^Measured by immunofluorescence. ^8^Measured by ELISA.

**Table 5:** Renal function in 50 patients with infections, ANCA positivity, and glomerular disease.

Feature	Positive patient number	Unreported patient number	Positive % of reported patients
Urine microscopy			
Hematuria	31 (E 20; O 21)	9 (E 7; O 2)	98
Dysmorphic RBCs, RBC casts	21 (E 5, O 16)		
Pyuria, WBC casts	6 (E: 3; O: 3)		
Proteinuria			
Increased urine protein excretion	35 (E 16; O 19)	15 (E 11; O 4)	100
Nephrotic proteinuria	10 (E 3; O 7)		29
Renal failure			
Elevated serum creatinine	39 (E 20; O 19)	9 (E 7; O 2)	95
Dialysis	9 (E 5; O 4)		18–24^1^

%: percent, RBC: red blood cell, WBC: white blood cell, E: endocarditis, O: other than endocarditis infection. ^1^Dialysis was not reported but may have been used in two patients with endocarditis and acute renal failure [36] and one patient with a chronic suppurative process who presented with a serum creatinine of 2,220 *μ*mol/L [110].

**Table 6:** Histological types of glomerular disease, serum complement levels, and ANCA specificity in 50 patients with infections, ANCA positivity, and glomerular disease.

Histological diagnosis	Number of patients	Percent of patients	Low serum complement	c-ANCA and/or PR3	p-ANCA and/or MPO	Other or Nonidentified ANCA
Pauci-immune GN, vasculitis	23	45	5/10 (38%)	5/23 (22%)	8/23 (35%)	10/23 (43%)
Postinfectious GN	19	38	15/17 (88%)	19/19 (100%)	1/19 (5%)	0
Anti-GMB GN	1	5	0	0	0	1 (100%)
Interstitial nephritis	5	10	4/5 (80%)	2/5 (40%)	3/5 (60%)	0
Acute tubular necrosis	4	8	2/3 (67%)	2/4 (50%)	2/4 (25%)	1/4 (25%)
Other histology^1^	2	2	1/1 (100%)	1/2 (50%)	1/2 (50%)	
Nonidentified GN^2^	7	12	3/3 (100%)	4/7 (57%)	4/7 (57%)	0

GN: glomerulonephritis, GBM: glomerular basement membrane. ^1^One each, focal glomerulosclerosis, chronic sclerosing glomerulonephritis. ^2^Immunofluorescence and electron microscopy findings were not reported. The differentiation between pauci-immune and postinfectious glomerulonephritis was not feasible from only the light microscopy picture.

**Table 7:** Treatment and outcomes of 50 patients with development of ANCA positivity and glomerulonephritis during the course of infectious episodes.

Treatment method	*N* (%)	Outcome	*N* (%)
Pauci-immune glomerulonephritis^1^	23		
Antibiotics	10 (43)	Improvement/recovery	17 (74)
Corticosteroids	22 (96)	Death	3 (13)
Cyclophosphamide	17 (74)	Not reported	3 (13)
Plasma exchange	2 (9)		
Interferon-a2b	1 (4)		
Aortic valve replacement	1 (4)		
Postinfectious glomerulonephritis^1^	19		
Antibiotics	17 (89)	Improvement/recovery	14 (74)
Corticosteroids	11 (58)	Dialysis	3 (16)
Cardiac valve replacement	3 (16)	Death	5 (26)
Atrioventricular shunt removal	3 (16)		
Ventricular septal defect repair	1 (5)		
Intravenous immunoglobulins	1 (5)		
Plasma exchange	1 (5)		
Interferon-a2b	1 (5)		
Cyclophosphamide	1 (5)		
Other types of renal disease	9	—	—
Antibiotics	8 (89)	Improvement/recovery	6 (67)
Corticosteroids	2 (22)	Chronic renal failure	2 (22)
Aortic valve replacement	1 (11)	Death	1 (11)
Cyclophosphamide	1 (11)		

^1^Including two patients with dual glomerulopathy [120, (a) and (b)].
